# Evaluation of compact pulsed lasers for two-photon microscopy using a simple method for measuring two-photon excitation efficiency

**DOI:** 10.1117/1.NPh.10.4.044303

**Published:** 2023-11-14

**Authors:** Samir Saidi, Matthew Shtrahman

**Affiliations:** a University of California, San Diego, Shu Chien-Gene Lay Department of Bioengineering, La Jolla, California, United States; b University of California, San Diego, Department of Neurosciences, La Jolla, California, United States

**Keywords:** two-photon microscopy, ultrashort pulsed lasers, fiber lasers, fluorescence excitation efficiency, pulse quality

## Abstract

**Significance:**

Two-photon (2p) microscopy has historically relied on titanium sapphire pulsed lasers that are expensive and have a large footprint. Recently, several manufacturers have developed less expensive compact pulsed lasers optimized for 2p excitation of green fluorophores. However, quantitative evaluation of their quality is lacking.

**Aim:**

We describe a simple approach to systematically evaluate 2p excitation efficiency, an empiric measure of the quality of a pulsed laser and its ability to elicit 2p induced fluorescence.

**Approach:**

By measuring pulse width, repetition rate, and fluorescence output, we calculated a measure of 2p excitation efficiency η, which we compared for four commercially available compact pulsed lasers in the 920 to 930 nm wavelength range.

**Results:**

2p excitation efficiency varied substantially among tested lasers. The Coherent Axon exhibited the best 2p excitation efficiency (1.09±0.03), exceeding that of a titanium sapphire reference laser (defined to have efficiency = 1). However, its measured fluorescence was modest due to its long pulse width. Of the compact lasers, the Toptica Femtofiber Ultra exhibited the best combination of measured fluorescence (0.75±0.01) and 2p excitation efficiency (0.86±0.01).

**Conclusions:**

We describe a simple method that both laser developers and end users can use to benchmark the 2p excitation efficiency of lasers used for 2p microscopy.

## 1 Introduction

Two-photon (2p) fluorescence microscopy is a laser-scanning microscopy method that provides excellent optical sectioning through thick and highly scattering samples, including tissue within living animals. 2p excitation of fluorescent molecules is a low probability event, which requires concentration of photons in space (tight focus) and time (pulsed light source). These properties restrict fluorescence generation to a highly localized focal volume [∼500 attoliters (5E-19 L) using a 920 nm light source and a 0.8 numerical aperture (NA) water immersion objective]^1^ that allows optical sectioning without requiring a pinhole. As a result, 2p fluorescence allows for efficient detection of fluorescence emission, requires modest average excitation power, and permits chronic imaging of biological samples over minutes to months.^2^ This form of microscopy has found particular interest in the neuroscience field where it has been adapted to record calcium dynamics^3^^,^^4^ and neurotransmitter release (Glu, DA, and GABA)^5^^–^^7^ with subcellular resolution in thick brain slices and deep within the brains of behaving animals.

Perhaps the largest barrier to the wide adoption and routine use of 2p microscopy is the acquisition of a suitable light source for 2p excitation. 2p microscopy relies on ultrashort (∼100 femtosecond) pulsed laser technology. The most abundant and optically stable genetically encoded fluorescent probes available to researchers are engineered from green fluorescent protein, which has a 2p excitation peak in the range of 900 to 930 nm.^8^^,^^9^ Until recently, the best available lasers suitable for efficient 2p excitation of these fluorophores were wavelength tunable titanium sapphire (Ti:Sa) lasers.^10^^,^^11^ While modern Ti:Sa lasers offer excellent beam quality and stability, they are prohibitively expensive for many laboratories, take up a large amount of both table and floor space, and require regular maintenance of the liquid cooling system, which produces a noticeable amount of audible noise. Recently, several laser manufacturers have developed air-cooled fiber and solid state lasers for 2p microscopy, which are less expensive and substantially more compact than Ti:Sa lasers. Unlike Ti:Sa lasers, these devices produce pulsed laser light at only a single fixed wavelength and have only recently become available in the highly sought after wavelength range of 920 to 930 nm with pulse durations of 150 fs or less, making them ideal for 2p microscopy of green fluorophores. However, quantitative evaluation of the performance of these lasers is lacking, particularly in comparison to Ti:Sa laser technology, which remains the gold standard in the field.

One measure of performance that is important for 2p microscopy in biological tissue, but is rarely measured, is 2p fluorescence excitation efficiency. In addition to fluorescence generation, laser light can result in tissue heating^12^^,^^13^ and formation of free radicals and other toxic species,^14^ all of which can damage and alter the physiology of biological tissues. In many cases, toxin formation and tissue damage grow faster with increasing laser power than fluorescence excitation, limiting laser powers that can be used in biological experiments.^14^ Optimizing the pulse quality and limiting spurious pulses and other power fluctuations minimizes the amount of potentially harmful energy that is deposited into tissue that does not result in maximal 2p excitation. This is a particularly important consideration when imaging dynamic signals in deep and highly scattering tissue, such as in the brain, where both generation and detection of adequate 2p fluorescence in a biologically relevant time window is challenging. Also, as the field moves toward wider adoption of 2p fluorescence microscopy,^15^ second-harmonic generation,^16^ and other nonlinear optical approaches^17^^,^^18^ for imaging in human subjects and patients, researchers will need to work diligently to maximize 2p excitation efficiency, thus minimizing toxicity.

Manufacturers of ultrashort pulsed lasers typically provide a test report that includes the repetition rate, pulse duration, and a measure of beam quality or spatial profile. Rarely, manufacturers will provide an indirect measure of pulse quality using techniques, such as frequency-resolved optical gating (FROG).^19^ The instrumentation required for these FROG measurements is expensive and complex and often impractical for the end user. In contrast, we describe a direct measurement of 2p fluorescence excitation efficiency, which is an empiric measure quantifying the amount of fluorescence generated by a pulsed laser independent of pulse width and repetition rate. This metric reflects laser pulse quality (see Sec. 2), among other factors, and is inexpensive and simple and captures an important metric for the microscopist. Surprisingly, this measurement is not provided by manufacturers and is rarely performed by end users.

In this study, we systematically tested available compact ultrashort pulsed lasers with wavelengths in the range of 920 to 930 nm and average power exceeding 500 mW for their fluorescence excitation efficiency as compared to a Ti:Sa laser. While we identify which of the lasers performed best in our testing, we also provide a simple protocol for laser manufacturers and investigators to perform their own analysis of fluorescence excitation efficiency to push the limits of what is achievable for compact ultrashort pulsed lasers in the future.

## 2 Pulsed Laser Background

The fluorescence generated by 2p excitation at any given time is directly proportional to the square of the instantaneous laser power. Assuming that all pulses generated by the laser are equivalent, the fluorescence generated by a laser at steady state can be calculated by the time-average of the power squared according to

$$F\propto R\int P_{\text{pulse}}^{2}(t)dt,$$ (1)

where F is fluorescence intensity, R is the laser repetition rate, and $P_{\text{pulse}}(t)$ is the power of a single pulse as a function of time. To bypass the challenges involved in measuring the time-dependent laser power profile over the fleeting duration of a single pulse, several assumptions are made.^20^ Assuming a square pulse with peak power $P_{pk}$ and duration $\tau_{p}$, Eq. (1) can be simplified to

$$F\propto R\cdot P_{pk}^{2}\cdot \tau_{p}.$$ (2)

We cannot easily measure the peak power of a pulse, but we can measure the average optical power generated by the laser. Keeping our assumption of identical square pulses, the average power is simply

$$P_{av}=R\cdot P_{pk}\cdot \tau_{p}.$$ (3)

**Fig. 1:**
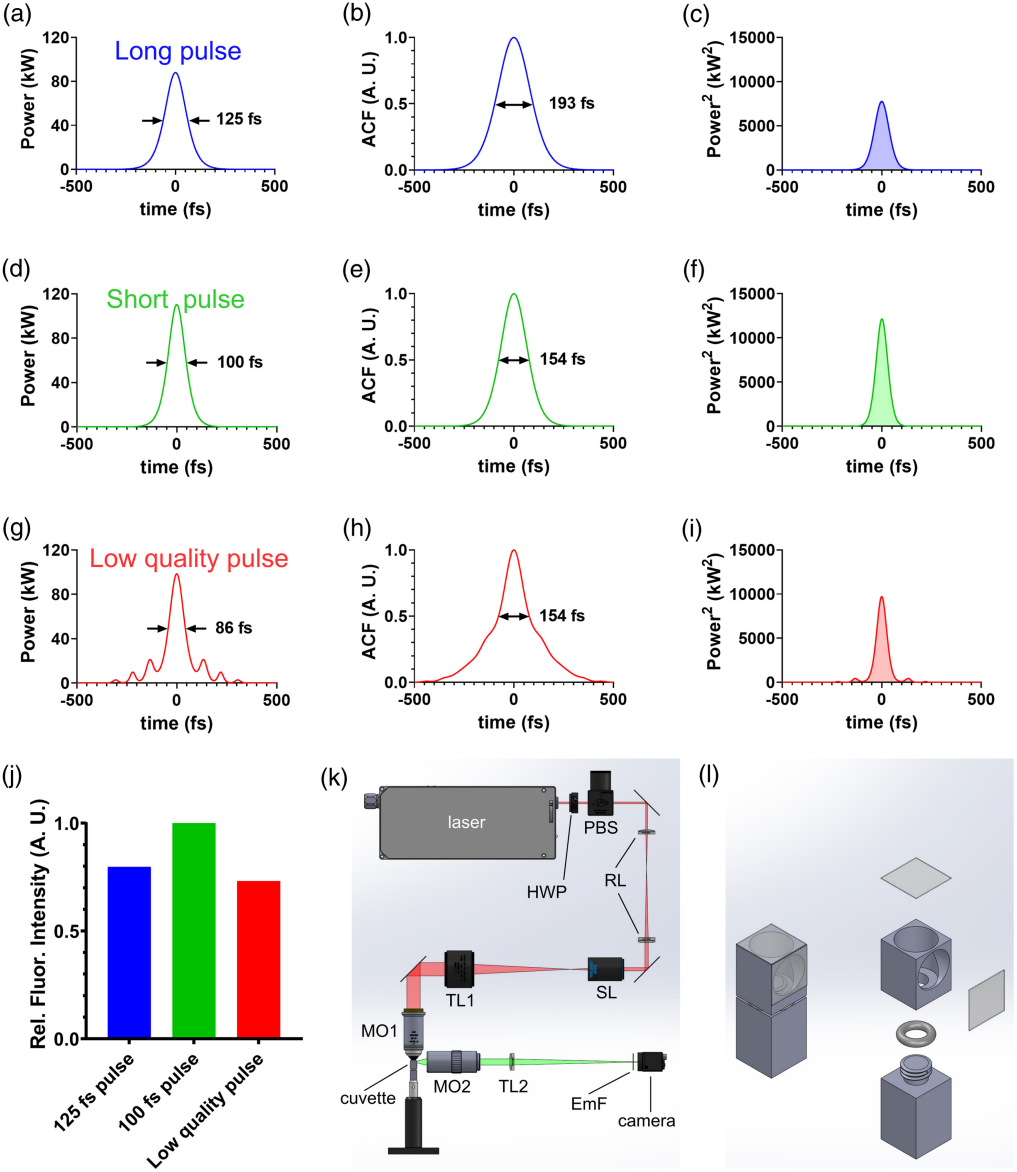
Pulse shape affects fluorescence intensity. (a), (d), and (g) The pulse profiles (power as function of time) of three different theoretical pulsed lasers are shown. All three lasers have identical pulse energy of 12.5 nJ and average power of 1 W at a repetition rate of 80 MHz. (b), (e), and (h) ACFs of the pulses are calculated from the first column. (c), (f), and (i) The square of the pulse profiles (${\text{power}}^{2}$ as a function of time) from the first column are shown. The area under these curves is directly proportional to fluorescence intensity, which is displayed for all three lasers in the bar graph in panel (j). The first two rows represent lasers with an ideal ${\mathrm{sech}}^{2}$ pulse shape with pulse durations of 125 and 100 fs, respectively. The third row represents a laser with a low quality pulse, with a significant fraction of the pulse energy outside of the central peak. Similar to the pulse in panel (d), this pulse’s autocorrelation has a width of 154 fs. Based solely on measurements of average power, repetition rate, and autocorrelation width, one would assume the fluorescence output from lasers 2 and 3 to be equivalent [see Eq. (4)]. However, as shown in panel (j), laser 3 produces less fluorescence than both lasers 1 and 2. Fluorescence from lasers 1 and 2 differs by a factor of 1.25, as predicted by the ACFs. (k) Optical setup for measuring the 2p fluorescence efficiency for each laser. The cuvette is filled with fluorescent dye, the laser is focused through a thin glass window on top (excitation light path shown in red), and the generated fluorescent spot is imaged onto the camera through the side window (emission light path shown in green). (l) Assembled and exploded views of the stainless steel cuvette assembly. (a)–(i) Adapted from Ref. 21. HWP, half-wave plate; PBS, polarizing beamsplitter; RL, achromatic doublet relay lenses; SL, microscope scan lens; TL1, microscope tube lens; MO1, 16× water immersion microscope objective; MO2, 20× long working distance air objective; TL2, achromatic doublet tube lens; EmF, green emission filter.

Rearranging to solve for the peak power and substituting into Eq. (2), we arrive at

$$F\propto \frac{P_{av}^{2}}{R\cdot \tau_{p}},$$ (4)

which allows us to predict how fluorescence intensity changes with measurable values of average power, repetition rate, and pulse duration.^20^ Although this relationship was derived using an assumption of a square pulse, this proportionality holds regardless of pulse shape so long as that shape remains consistent between lasers. In theory, a high quality pulsed laser with good 2p excitation efficiency will produce a pulse with a ${\mathrm{sech}}^{2}$, Gaussian, or similar shape with a prominent central peak. However, femtosecond lasers can exhibit distorted pulses with only a fraction of the laser power contained within the central peak and the remaining energy present within “side lobes” [Fig. 1(g)].^21^ To account for imperfections in the pulse shape and other spurious features of the laser that can affect fluorescence, we can introduce a dimensionless quantity η, for 2p excitation efficiency, that modifies Eq. (4):

$$F\propto \eta \cdot \frac{P_{av}^{2}}{R\cdot \tau_{p}}.$$ (5)

As discussed above, femtosecond laser pulses required for 2p microscopy are generally too short to measure directly. Therefore, the pulse duration $\tau_{p}$ is derived using an autocorrelator, which measures the autocorrelation function (ACF) of the pulse profile. The ACF integrates the product of the pulse profile and a time shifted version of itself. The ACF is always symmetric, regardless of the pulse profile. The ACF amplitude is normalized to 1 and has a width defined by the time it takes the ACF to decay to ½, termed the autocorrelation time. The measured ACF does not uniquely determine the original pulse profile, and in practice, a ${\mathrm{sech}}^{2}$ or similar pulse shape is often assumed. When laser manufacturers report pulse width, they typically divide the ACF width by a constant deconvolution factor to match the full width half max (FWHM) of the assumed pulse shape (1.54 for a ${\mathrm{sech}}^{2}$ pulse). This assumption of a ${\mathrm{sech}}^{2}$ pulse shape is arbitrary (see Sec. 3.1), but it is usually adequate if the pulse is of high quality, i.e., the vast majority of the pulse energy is confined within the central peak. If, however, the pulse produced by the laser is of low quality, the pulse’s peak power is not as high as presumed from the ACF because some of the pulse energy is lost in the side lobes and does not contribute meaningfully to the generation of 2p fluorescence. Therefore, a laser with a low quality pulse will generate less fluorescence than predicted from the measured average power, repetition rate, and ACF alone as compared to one with a high quality pulse.^21^ This effect is illustrated in Fig. 1. The first row depicts an ideal ${\mathrm{sech}}^{2}$ laser pulse, with $\tau_{p}=125\;\mathrm{fs}$ [Fig. 1(a)], and its ACF [Fig. 1(b)]. The second row depicts a pulse with the same pulse energy and shape, but a shorter pulse duration of $\tau_{p}=100\;\mathrm{fs}$ [Fig. 1(d)], and its ACF [Fig. 1(e)]. Assuming a constant repetition rate, measures of relative fluorescence output [Fig. 1(j)] can be obtained by squaring the pulses [Figs. 1(c) and 1(f)] and integrating over time (shaded area under the curve) as described in Eq. (1). Note that both the actual pulse duration and the ACF width of the second laser are 1.25× shorter than those of the first laser and that the fluorescence output of the second laser is 1.25× greater than that of the first laser, consistent with Eq. (4). Based on Eq. (4), one might expect that a shorter pulse duration (shorter ACF width) would always increase fluorescence output for a fixed average laser power and repetition rate. The third row depicts an example of a low quality pulse [Fig. 1(g)]. In this case, the ACF width is 154 fs [Fig. 1(h)]. Equation (4) predicts that the fluorescence output will be comparable to the output from laser 2. However, squaring and integrating the pulse reveals that the fluorescence output is actually lower than both lasers 1 and 2 [Fig. 1(j)], despite having the same or even shorter pulse duration than those lasers. Thus, another factor is required to account for poor pulse quality and other spurious effects that can degrade 2p fluorescence output.

## 3 Results

**Fig. 2:**
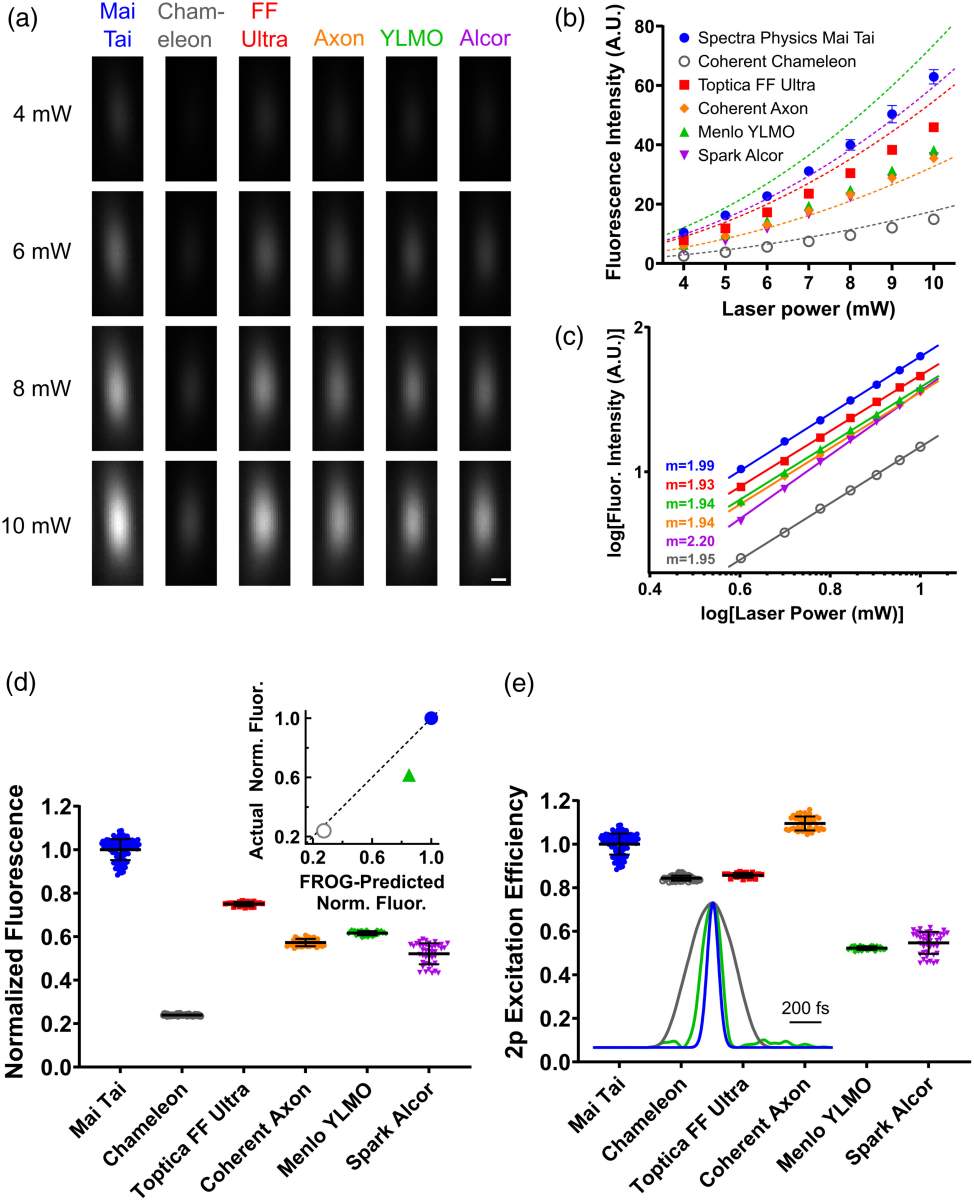
A simple measure of 2p excitation efficiency. (a) Representative images of the fluorescent spot captured by the camera in Fig. 1(k) for the different lasers tested at different incident power levels, normalized by exposure time (see Sec. 5.3). Scale bar=1  μm. (b) Measurements of fluorescence intensity $F_{\text{test}}$ are plotted for each laser tested. Dashed lines show expected fluorescence intensity $F_{\text{ideal}}$ when accounting for differences in repetition rate and pulse duration between the tested laser and the Mai Tai Ti:Sa reference laser [see Sec. 3.1 and Eq. (6) for details]. (c) Log–log plot of the data in panel (b) with slope values displayed, demonstrating that the fluorescence is generated with 2p excitation (m=2) without significant saturation or quenching. (d) Raw fluorescence intensity measurements across all power levels from panels (b) and (c) normalized to the average intensity produced by the Mai Tai reference laser at the same power level ($F_{\text{test}}/F_{\mathrm{ref}}$). Inset shows agreement between fluorescence predicted by the FROG measurement [see Sec. 5.5 and Fig. 2(e) inset] and measured fluorescence for a subset of the lasers. FROG-predicted fluorescence is plotted on the x-axis and measured fluorescence is plotted on the y-axis, with perfect agreements represented by the dashed line. Both axes are normalized to the values for the Mai Tai. Statistically significant differences (p<0.0001) exist between every pair of means (Dunnett’s T3 multiple comparisons test). (e) Fluorescence intensity ratios from panel (d) scaled for differences in repetition rate and pulse duration between the tested laser and the Mai Tai reference laser ($F_{\text{test}}/F_{\text{ideal}}$), which serves as a measure of 2p excitation efficiency [see Eq. (6)]. Computed p-values for differences between every pair of means is shown in Table 2 (Dunnett’s T3 multiple comparisons test). Inset shows pulse traces measured with FROG for three lasers (blue: Mai Tai, gray: Chameleon, and green: YLMO), demonstrating that pulse quality correlates qualitatively to 2p excitation efficiency. Error bars indicate standard deviation (not shown where standard deviation is smaller than marker size).

To compare the 2p fluorescence excitation efficiency of different pulsed lasers, we constructed a simple 2p microscope containing the same key optical elements as the excitation path of a typical microscope used for *in vivo* 2p imaging [Fig. 1(k)]. For each laser, the relay lenses were chosen to achieve the same excitation beam diameter, slightly overfilling the back aperture of a water immersion objective (Nikon CFI75 LWD 16X W) to achieve the full NA. Keeping the beam aligned with the optical axis, the laser beam was focused into a custom cuvette containing a green fluorescent solution [10  μM Atto 488, Fig. 1(l)]. The resulting fluorescent excitation volume was imaged from the side with another microscope objective onto a camera to capture its x-z profile [Figs. 1(k) and 2(a)]. The peak fluorescence intensity from each fluorescent spot was measured at laser powers ranging from 4 to 10 mW, which is shown in Fig. 2(b). To rule out saturation of fluorophores and quenching (observed at laser power levels beyond 10 mW), fluorescence intensity was plotted against average power for each laser on a log-log plot to ensure that the square relationship between these quantities, as described in Eq. (4), was preserved [Fig. 2(c)].

### 3.1 Two-Photon Excitation Efficiency Measurement

In addition to comparing the fluorescence intensity excited by each laser as a function of average power, we derived a measure of 2p excitation efficiency (η) for each laser tested, as introduced in Eq. (5). Using a Ti:Sa laser, the Spectra Physics Mai Tai, as a gold standard reference and defining it to have an efficiency of η=1, the 2p excitation efficiency of each tested laser was calculated using

$$\eta =\frac{F_{\text{test}}}{F_{\text{ideal}}}=F_{\text{test}}\cdot \frac{R_{\text{test}}\cdot \tau_{p,\text{test}}}{F_{\mathrm{ref}}\cdot R_{\mathrm{ref}}\cdot \tau_{p,\mathrm{ref}}}.$$ (6)

Here $F_{\text{test}}$ is the measured intensity of the fluorescent spot generated by the laser being tested at a given average power. $F_{\text{ideal}}$ is the expected fluorescence intensity of the test laser if it had the same 2p excitation efficiency (η) as the reference laser. This value is obtained by adjusting $F_{\text{ref}}$ for differences in the repetition rate and pulse duration of the two lasers using Eq. (4). As discussed in Sec. 2 and described in Fig. 1, if the reference laser has a pulse duration (or repetition rate) that is twice as long (twice as fast) as the test laser, $F_{\text{ideal}}$ should be two times greater than $F_{\mathrm{ref}}$. Thus, the ratio of $F_{\text{test}}/F_{\text{ideal}}$ provides a simple measure of 2p fluorescence efficiency.

The repetition rate R was measured by monitoring the output of a fast photodiode equipped with each laser. Each laser’s pulse duration $\tau_{p}$ was measured using an autocorrelator and assuming a ${\mathrm{sech}}^{2}$ pulse shape. Note that the assumption of ${\mathrm{sech}}^{2}$ pulse shape for reporting pulse duration is arbitrary and does not affect any of the results, which all depend on the $\tau_{p}$ ratios, but is used rather to maintain consistency with published manufacturer specifications. Values for the repetition rate and pulse duration of the Spectra Physics Mai Tai Ti:Sa reference laser, a Coherent Chameleon Ultra II Ti:Sa laser, and the four compact lasers are shown in Table 1. The normalized fluorescence intensity $F_{\text{test}}/F_{\mathrm{ref}}$ and 2p excitation efficiency η for each laser are plotted in Figs. 2(d) and 2(e), respectively. Due to a combination of factors (R, $\tau_{p}$, and η), the Mai Tai Ti:Sa reference laser produced more fluorescence for the same average power than any of the compact lasers tested [Fig. 2(d)]. Once adjustments are made for the repetition rate and pulse duration, one can see that the 2p excitation efficiency varies dramatically between the various lasers. Surprisingly, the 2p excitation efficiency of the Coherent Axon 920 (mean: 1.09 ± standard deviation: 0.03) actually exceeded that of the Spectra Physics Mai Tai reference laser [1.00±0.05, Fig. 2(e)] even though its measured (normalized) fluorescence $F_{\text{test}}/F_{\mathrm{ref}}$ (0.57±0.02, Fig. 2(d) was modest compared to the Ti:Sa laser (1.00±0.05), due to its long pulse duration. In contrast, the 2p excitation efficiency of the Menlo Systems YLMO laser (0.52±0.01) was significantly worse than that of the Spectra Physics Mai Tai reference laser, reflecting in part its poor pulse quality with prominent side lobes [see green FROG trace in inset of Fig. 2(e)] compared to the Ti:Sa lasers. The fluorescence predicted from the FROG trace correlated with measured fluorescence for the few lasers where this measurement was available [inset of Fig. 2(d)], consistent with the idea that pulse shape is a factor in determining 2p excitation efficiency. It was found, however, that the FROG trace did not fully explain the variations in fluorescence observed. For the YLMO, the FROG trace overestimated the actual fluorescence by 42%, and for the Chameleon, the FROG trace overestimated the actual fluorescence by 18%, when using the Mai Tai as a reference. Thus, the more direct 2p excitation efficiency measurement carried out in this study appears to account for factors other than pulse quality as measured by FROG. Of the tested compact lasers, the Toptica Femtofiber Ultra 920 had perhaps the best combination of measured fluorescence $F_{\text{test}}/F_{\mathrm{ref}}$ (0.75±0.01), due to its favorable repetition rate and pulse duration and 2p excitation efficiency η (0.86±0.01).

**Table 1:** Measured repetition rate and pulse duration of the Ti:Sa reference laser (Spectra Physics Mai Tai) and each tested laser. These data are shown in graphical form in Fig. S4 in the Supplementary Material.

	Spectra Physics Mai Tai	Coherent Chameleon Ultra II	Toptica Femtofiber Ultra 920	Menlo Systems YLMO 930	Coherent Axon 920	Spark Alcor 920-2
Repetition rate (MHz)	80.78	80.36	80.51	50.61	80.13	78.93
Pulse duration (fs)	82	291	94	111	158	88
Center wavelength (nm)	920	920	920	930	920	920

**Table 2:** Computed p-values for differences between means of 2p excitation efficiency [see Fig. 2(e)] using Dunnett’s T3 multiple comparisons test.

	Mai Tai	Chameleon	FF Ultra	YLMO	Axon	Alcor
Mai Tai	—	<0.0001	<0.0001	<0.0001	<0.0001	<0.0001
Chameleon	—	—	0.0018	<0.0001	<0.0001	<0.0001
FF Ultra	—	—	—	<0.0001	<0.0001	<0.0001
YLMO	—	—	—	—	<0.0001	0.0575
Axon	—	—	—	—	—	<0.0001
Alcor	—	—	—	—	—	—

**Fig. 3:**
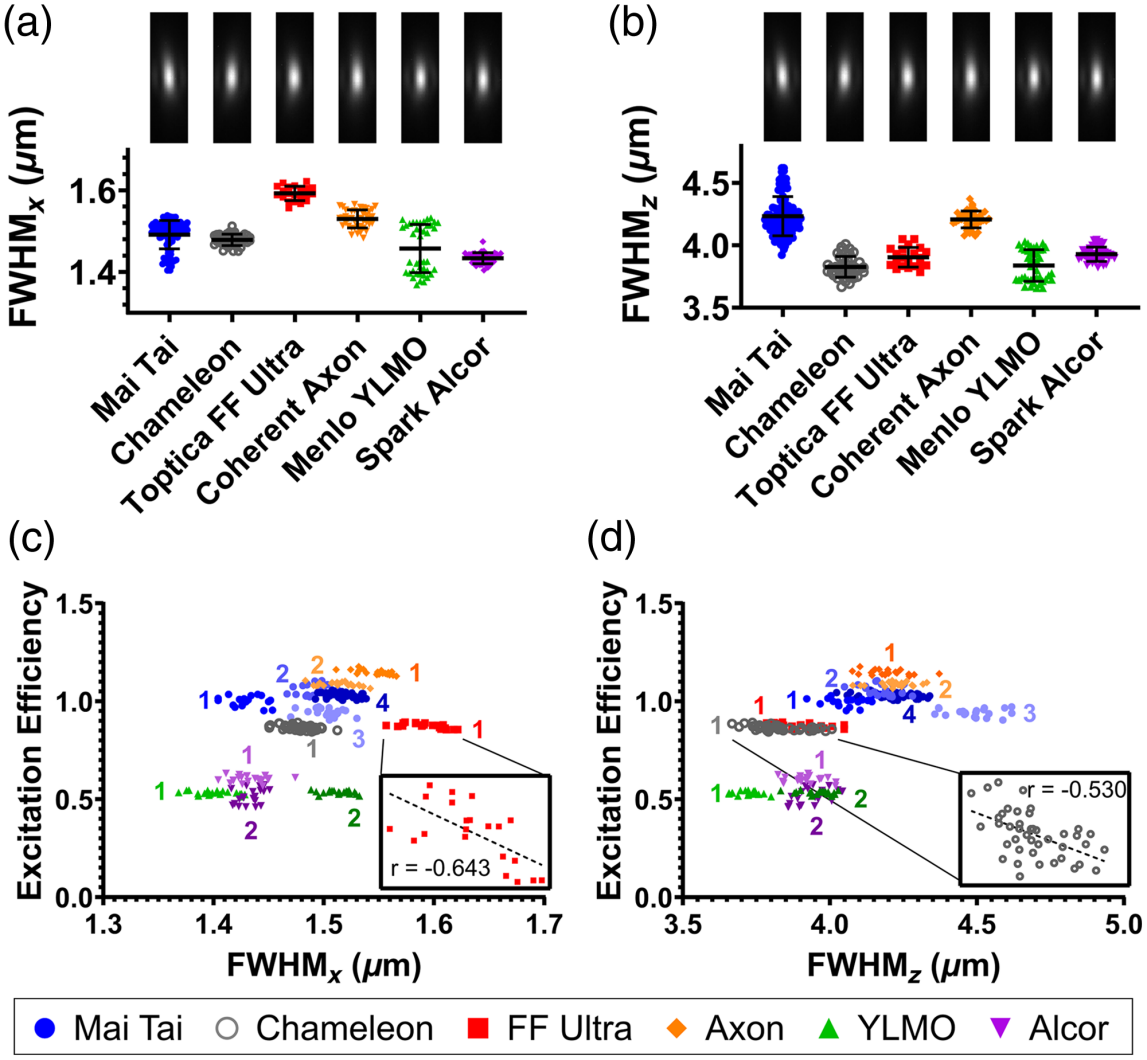
Differences in pulse quality cannot be explained by variation in laser spot size. Measurements of the FWHM of the excitation volume in the (a) lateral and (b) axial dimensions for each laser across six different power levels are shown below representative excitation volume images. Note that the same set of images is used in both panels (a) and (b). Error bars indicate standard deviation. (c), (d) 2p excitation efficiency [see Eq. (6), Fig. 2(e)] is not dependent on the size of the excitation volume. Different shading and numbering are used to denote multiple data-collection sessions on different days with laser realignment in between (three sessions for the Mai Tai; 2 for the YLMO, Axon, and Alcor; and 1 for the Chameleon and FF Ultra). (c) Radial and (d) axial spot dimensions exhibited a slight positive correlation with 2p excitation efficiency across different lasers and sessions ($r_{\text{radial}}=0.487$ and $r_{\text{axial}}=0.677$, respectively). Slight negative correlations are seen across the vast majority of single data-collection sessions, with examples shown in insets. All single session correlation coefficients are provided in Tables S1 and S2 in the Supplementary Material.

To verify that the measured differences in 2p excitation efficiency were not due to changes in the optical system or alignment, the sizes of the excitation volumes produced by each laser were measured and compared. If the measurements of $F_{\text{test}}/F_{\mathrm{ref}}<1$ that we observed were due to aberrant foci, one would expect a negative correlation between 2p excitation efficiency and spot size across lasers. Figure 3 shows that spot sizes instead exhibited a slight positive correlation across lasers for these values (r=0.487 in the x dimension and r=0.677 in the z dimension) [Figs. 3(c) and 3(d)]. Thus, differences in 2p excitation between lasers could not be explained by differences in the radial or axial dimension of the laser focus. As expected, slight negative correlations are seen between 2p excitation efficiency and spot size across measurements during a single session for a given laser [see insets of Figs. 3(c) and 3(d) and Tables S1 and S2 in the Supplementary Material]. These variations for a single laser are minute, however, compared to the difference in 2p excitation efficiency between the different lasers.

## 4 Discussion

Here we describe a simple method for comparing the fluorescence excitation efficiency of ultrashort pulsed lasers used in 2p microscopy. Because fluorescence emission is a random Poisson process, maximizing the fluorescent signal is desirable in order to maximize the signal-to-noise ratio. While this can be achieved by increasing laser power, tissue toxicity grows with increasing levels of both average and instantaneous power.^13^^,^^14^ Therefore, it is vital to identify lasers that maximize fluorescent signal for given average and instantaneous powers. Unfortunately, average power, pulse duration, and repetition rate alone are not sufficient to predict the relative fluorescence produced by different pulsed lasers. It is clear from our measurements that there are other factors, including pulse quality, that determine fluorescence excitation but are difficult to measure directly. For the few lasers where we could measure FROG traces, the pulse quality appeared to predict measured fluorescence to a large extent. However, the FROG measurement does not appear to be an adequate substitute for measuring 2p excitation efficiency [inset of Fig. 2(e)], which is simpler and accounts for all variables that can degrade fluorescence excitation.

Depending on whether one is concerned with optimizing fluorescence for a given average laser power or maximizing 2p excitation efficiency (see Sec. 3.1 for details), two frontrunners emerge from the tested compact ultrashort pulsed lasers. The Toptica Femtofiber Ultra 920 had the highest generated fluorescence for a fixed amount of average laser power [Fig. 2(d)], due to having a combination of relatively short pulse duration and 2p excitation efficiency. When adjusting the measured fluorescence generated by each laser for repetition rate and measured pulse duration, the Coherent Axon 920 laser had the highest 2p excitation efficiency, exceeding even that of the Ti:Sa lasers.

This study provides important guidance when selecting pulsed lasers for 2p microscopy, but it also has some limitations. While we can assess the performance of the individual laser units that we received for testing, we cannot state to what extent these results generalize across their production line. For all four of these compact laser models, we tested at least two different units and chose to publish the results from the best performing unit. For every laser, we observed significant differences in performance between different units of the same model, including some instances where the lasers exhibited poor or anomalous behavior, including stability issues that precluded accurate measurements. This motivated the decision to only publish the results from the best performing unit of each model. We also did not quantify the long-term reliability, ease of use, feature availability, and other metrics of importance to the end user of these lasers. Finally, as indicated by the experience above, the laser manufacturers are constantly iterating their products, so the typical performance of these lasers will likely improve in the future.

It is important to note that this work is intended to serve as a simple protocol that developers and manufacturers of pulsed lasers can follow to benchmark the fluorescence 2p excitation efficiency of their lasers in both the development and quality control stages. Perhaps more importantly, we also hope to inspire other 2p microscopists working in the neurosciences and other fields to carry out similar tests to characterize their own lasers.

## 5 Appendix: Methods

### 5.1 Cuvette Design and Assembly

To measure 2p fluorescence efficiency, a custom stainless steel cuvette filled with 10  μM Atto 488 dye and capped with a No. 1 cover glass was constructed in a manner such that no air gap between the cover glass and the solution was introduced [see Fig. 1(l); Code, Data, and Materials Availability section for computer-aided design (CAD) files and drawings]. The cuvette was comprised of a 10  mm×10  mm×12.5  mm stainless steel block with Ø 9 mm holes bored into the top and side to form the cavity for the solution and to form the excitation and viewing windows. A Unified National Fine (UNF) 1/4-28 threaded hole was formed in the bottom for mating with the base. Two 10  mm×10  mm squares were cut from No. 1 cover glass using a diamond scribe. The pieces of cover glass were bonded to the excitation and viewing windows with optical adhesive (Norland Products NOA 81) and cured using ultraviolet (UV) light. The base of the cuvette was comprised of a 10  mm×10  mm×15  mm stainless steel block with a protruding UNF 1/4-28 threaded rod for mating with the cuvette. A size −008 soft Viton O-ring (McMaster-Carr 1284N108) was slipped over the rod to ensure a watertight seal when screwed into the threaded hole of the cuvette. A Unified National Coarse (UNC) 8-32 threaded hole was formed in the bottom face of the base, opposite the rod, for mating to a standard 1/2″ optical post (e.g., Thorlabs TR series).

To fill the cuvette, it was inverted and slightly overfilled with 10  μM Atto 488 solution using a transfer pipette. The cuvette base was then carefully but tightly threaded into the cuvette, ensuring minimal formation of air bubbles. The filled cuvette was stored in an inverted position between experiments to prevent the migration of air bubbles to the excitation window.

### 5.2 Measurement of Generated Fluorescence

The beams of the reference laser (Spectra Physics Mai Tai HP with DeepSee), the second Ti:Sa laser (Coherent Chameleon Ultra II), and each of the four compact lasers (Toptica Femtofiber Ultra 920, Coherent Axon 920, Menlo Systems YLMO 930, and Spark Alcor 920-2) were expanded and collimated, using a pair of achromatic doublet lenses and a standard 2p microscope scan lens (Thorlabs SL50-2P2) and tube lens (Thorlabs TTL200MP), to just overfill a water immersion microscope objective (Nikon CFI75 LWD 16X W). The objective was lowered into a drop of water on the cuvette’s coverslip to focus the beam into the dye and generate a volume of excited fluorescence. Fluorescence was imaged onto a camera (FLIR BFLY-PGE-31S4M-C) through a No. 1 cover glass window on the side of the cuvette using a long working distance objective (Mitutoyo Plano Apo Infinity Corrected LWD 20X) and an f=200  mm achromatic doublet tube lens (Thorlabs AC254-200-A). An IR-blocking filter was placed in front of the camera to block out laser light. Except for the Chameleon Ultra II Ti:Sa laser, which does not have a group delay dispersion (GDD) compensator, each laser’s built-in GDD compensator was tuned until the highest single pixel value in the image was maximized. See Fig. 1(k) for the schematic.

Each laser tested was manually swapped and aligned into the optical system. The same laser was sometimes realigned multiple times for multiple imaging sessions [see Figs. 3(c) and 3(d)]. Because the different compact lasers were tested in some cases several months apart, the fluorescence measurement with the Mai Tai Ti:Sa reference laser was repeated whenever a new subset of the compact lasers was measured to verify the stability of the fluorescence from the cuvette over time (see Fig. S2 in the Supplementary Material).

### 5.3 Image Capture and Normalization

For each image of the excitation volume taken, the camera exposure was adjusted in the range of 600 ms to 5 s so that the intensity of the brightest pixel was maximized without saturating. This resulted in different exposure levels across different lasers and different power levels. Each image was stored as a matrix of 16-bit unsigned integers (12-bit pixel depth scaled to 16 bits). To normalize images with different exposure levels for direct comparison, each image was first converted to double precision. Then the mean value of the background was calculated and subtracted from the image. Finally, the new image was divided by the exposure value in milliseconds. The linearity of the camera and this conversion was confirmed by imaging the same fluorescent spot with a series of different exposure times (Fig. S1 in the Supplementary Material). The value of the brightest pixel in each resulting image was used to quantify intensity. The excitation volume profile measurements were taken along the row and column of the brightest pixel in each image. Image processing and calculations were performed using custom routines in MATLAB.

To compute values for normalized fluorescence [plotted in Fig. 2(d)], every data point across all lasers and all power levels was divided by the fluorescence value predicted by the log–log regression of the Mai Tai at the same amount of laser power. This allows for comparison of fluorescence from each laser independent of power level and brings the average value of fluorescence from the Mai Tai reference laser to 1, facilitating the calculation of fluorescence ratios between lasers.

### 5.4 Pulse Duration and Repetition Rate Measurements

For each laser, except the Coherent Chameleon Ultra II, a small fraction of the beam intensity exiting the laser head was reflected using a cover glass and directed into an autocorrelator (APE Mini PD). A 1.5 ps scan length was used in FRINGES mode with an averaging factor of 8 and smoothing turned off. Sensitivity and gain were adjusted to achieve high dynamic range without saturating. The autocorrelator calculated the pulse duration from the autocorrelation trace by measuring the width of the ACF when it decays to ½ its amplitude (autocorrelation time) and dividing the value by the deconvolution factor for a ${\mathrm{sech}}^{2}$ pulse (1.54). Each laser’s group delay dispersion compensator was adjusted until the peak on the autocorrelator display was maximized and the displayed pulse duration was minimized. The reported pulse duration was then recorded.

The pulse width of the Coherent Chameleon Ultra II was measured using an FROG device (Swamp Optics GRENOUILLE 10-100-USB). Because there is no GDD compensation on the Chameleon, the beam was sent into the device after being propagated through the optical system used for fluorescence measurements (see Sec. 5.5 for details) to maintain a consistent GDD between fluorescence and pulse width measurements. To remain consistent with the pulse width measurements for the other lasers, the pulse width of the Chameleon Ultra II was calculated using the FROG device’s autocorrelation measurement and dividing by the ${\mathrm{sech}}^{2}$ deconvolution factor of 1.54.

Each laser came equipped with a fast photodiode placed in the path of a reflected portion of the beam. To measure the repetition rate of each laser, its photodiode output was connected to a digital oscilloscope (Tektronix TDS1001B), which automatically calculated the frequency of the recorded trace.

### 5.5 FROG Measurements and Calculations

FROG measurements were performed on a subset of the lasers tested in this study (Spectra Physics Mai Tai, Coherent Chameleon Ultra II, and Menlo Systems YLMO 930) to compare actual fluorescence measurements to the fluorescence predicted by the pulse shape and duration. To capture the effects of GDD and each laser’s built-in GDD compensation (or lack thereof in the case of the Chameleon) on the pulse, the laser beam was sent into a FROG device (Swamp Optics GRENOUILLE 10-100-USB) after being propagated through the simple microscope optical system [Fig. 1(k)]. Because the device requires a collimated input beam, the convergent beam exiting the microscope objective was first collimated with a 0.83 NA aspheric lens (Edmund Optics 67-257) before being coupled into the FROG device. Note that the water immersion normally used with the Nikon 16× objective was not used in the FROG measurements. To keep the beam at a reasonable diameter (∼4  mm) for the FROG device and avoid aberrations, the two initial achromatic doublets in the system were switched for achromatic doublets with different focal lengths to reduce the initial beam magnification and underfill the microscope objective. While this greatly lowered the effective NA of the microscope objective, swapping the achromatic doublets and adding the aspheric lens had minimal effect on the overall GDD of the system.

The normalized fluorescence (described in paragraph 2 of Sec. 5.3) was plotted against the fluorescence ratio predicted by FROG [see inset of Fig. 2(d)]. The FROG-predicted fluorescence ratio was calculated directly from the FROG pulse trace, which is independent of laser power and has an amplitude of 1 in arbitrary units. First, a constant laser power level of 1 W was assumed to simplify calculations. The FROG pulse trace was integrated using trapezoidal sums. Every point in the pulse trace was then divided by this integral and multiplied by the pulse energy for that laser at 1 W (1 W divided by the repetition rate) to yield a pulse trace with an integral equal to the energy of a single pulse at 1W average power. Then this power-normalized pulse trace was squared and integrated using trapezoidal sums, and this value was multiplied by the laser repetition rate to yield a value for the predicted 2p fluorescence of each laser, as in Eq. (1). Finally, this predicted value for each laser was normalized to the corresponding value for the Mai Tai reference laser to yield the FROG-predicted fluorescence ratio. See Fig. S3 in the Supplementary Material inset for representative FROG pulse traces from each of the lasers measured.

### 5.6 Wavelength Dependence of Fluorescence

All of the compact 2p lasers tested have a nominal peak wavelength of 920 nm, except for the Menlo Systems YLMO 930, which has a peak wavelength of 930 nm. To ensure that this difference in wavelength would not affect the amount of excited fluorescence and the calculated pulse quality, the methods in Secs. 5.1–5.4 were repeated with the Mai Tai Ti:Sa laser tuned between 920 and 930 nm. The fluorescence intensity was measured at each location and normalized by the pulse width at each location to isolate the wavelength dependence of 2p excitation efficiency of the Atto 488 dye. There was no significant difference in fluorescence intensity generated among the different wavelengths [One-way analysis of variance (ANOVA), p=0.26, see Fig. S3 in the Supplementary Material].

## Data Availability

All data as well as CAD files in STEP and Solidworks file formats and engineering drawings in PDF format for the custom cuvette (Sec. 5.1) are provided for download at https://github.com/shtrahman-lab/2p-Efficiency.
