# Supplementary material for: Evaluation of compact pulsed lasers for two-photon microscopy using a simple method for measuring two-photon excitation efficiency
Source: Neurophotonics. 2023 Nov 14;10(4):044303. doi: 10.1117/1.NPh.10.4.044303 (PMC10704185; doi:10.1117/1.NPh.10.4.044303)
Supplement: Supplementary file 1 [file NPh_010_044303_SD001.pdf]

Supplementary Material

Table S1 Pearson correlation coefficients (r) for the correlation between 2p excitation efficiency and the radial (x) dimension of the excitation volume are shown, as discussed in Fig. 3. Ten of the 12 values are negative.

	Spectra Phsyics Mai Tai	Coherent Chameleon	Toptica Femtofiber Ultra	Coherent Axon	Menlo Systems YLMO	Spark Alcor
Session 1	-0.201	-0.473	-0.643	-0.687	-0.389	0.405
Session 2	0.027	-----	-----	-0.434	-0.255	-0.055
Session 3	-0.237	-----	-----	-----	-----	-----
Session 4	-0.266	-----	-----	-----	-----	-----

Table S2 Pearson correlation coefficients (r) for the correlation between 2p excitation efficiency and the axial (z) dimension of the excitation volume are shown, as discussed in Fig. 3. Nine of the 12 values are negative.

	Spectra Phsyics Mai Tai	Coherent Chameleon	Toptica Femtofiber Ultra	Coherent Axon	Menlo Systems YLMO	Spark Alcor
Session 1	0.208	-0.530	-0.534	-0.388	-0.192	0.514
Session 2	-0.301	-----	-----	0.247	-0.292	-0.258
Session 3	-0.052	-----	-----	-----	-----	-----
Session 4	-0.247	-----	-----	-----	-----	-----

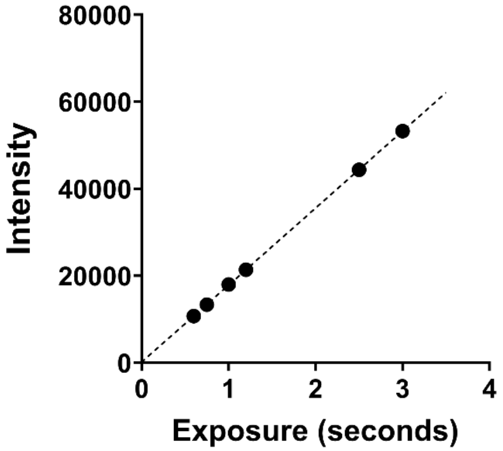

Fig. S1 *Camera pixel intensity is proportional to exposure time.* To confirm linearity of the camera exposure time and fluorescence intensity measurements, the cuvette fluorescence measurement (see Fig. 1k) was performed with different camera exposure values using the Mai Tai as the excitation source at 4 mW of laser power. Exposure values used correspond to the exposure values used to measure fluorescence data from the Mai Tai at the tested power levels of 4 mW (3 seconds exposure) to 10 mW (0.6 seconds exposure). n = 3 at each exposure level, standard deviation is smaller than marker size, R^2 = 0.9998.

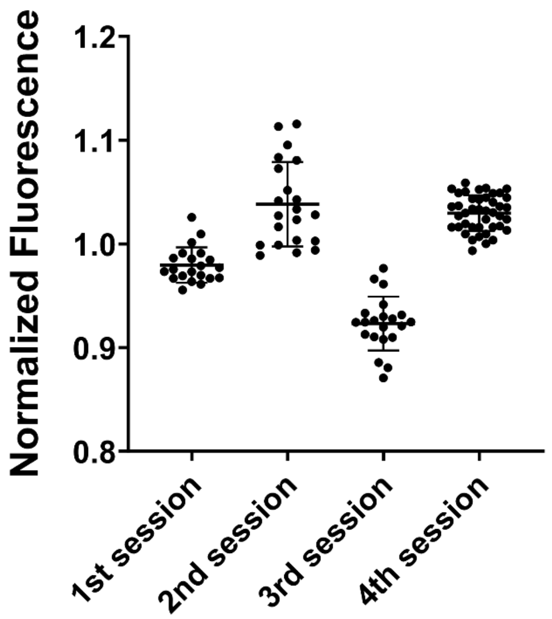

Fig. S2 *The fluorescence of the sample cuvette remained stable with no detectable loss over the duration of the experiment.* The fluorescence measurement with the Mai Tai Ti:Sa reference laser was repeated at 4 sessions spaced months apart throughout the duration of this experiment to verify that there was no detectable loss in fluorescence from the cuvette over time. While there is a small significant difference, there is no trend over time, and the mean value of any one session does not differ from the overall mean by more than 8%.

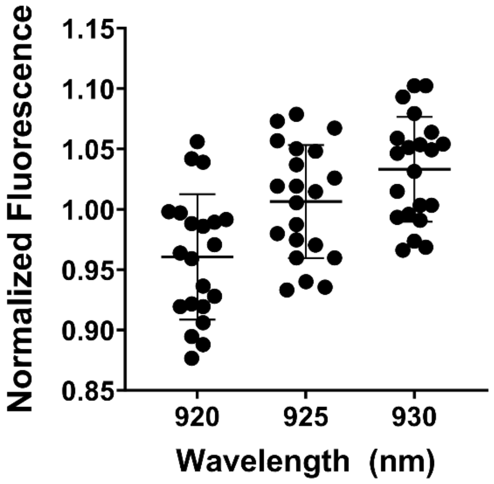

Fig. S3 *Fluorescence excitation efficiency is not dependent on wavelength in the 920 to 930 nm range.* To determine whether the difference in wavelength (930 vs 920 nm) was at all responsible for the inferior performance of the YLMO 930 laser, the cuvette fluorescence measurement (see Fig. 1k) was performed with the Mai Tai tuned to different wavelengths while maintaining 10 mW of laser power coming out of the objective. At each wavelength, the pulse width was measured with an autocorrelator and multiplied with the measured fluorescence intensity to arrive at the normalized fluorescence value, thus isolating the effect of the laser's center wavelength on fluorescence excitation. Although there appears to be a trend, there is no significant difference between the fluorescence excited at the different wavelengths (One-way ANOVA, $p = 0.26$).

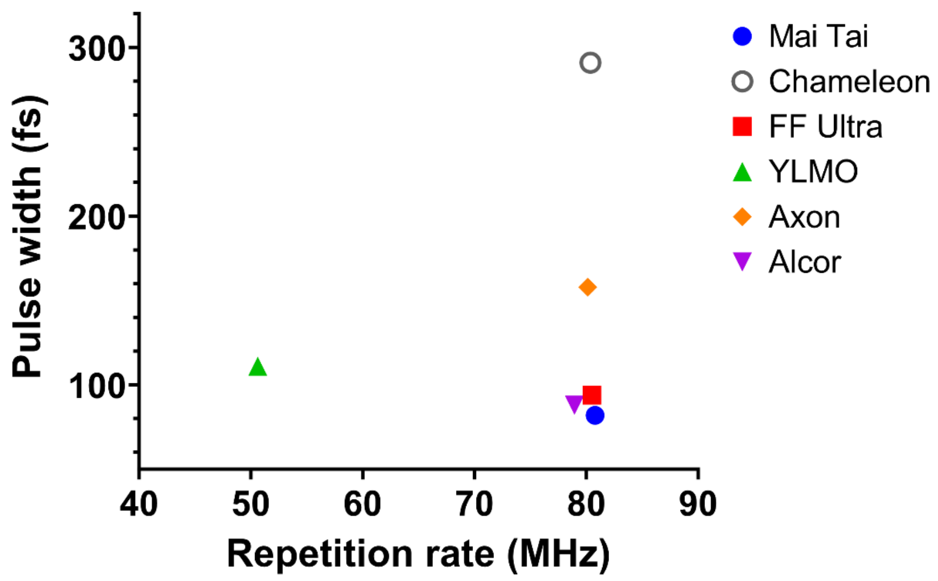

Fig. S4 Scatter plot of repetition rates and pulse widths for all lasers, as reported in Table 1.
